# 

**Published:** 1896-07

**Authors:** 


### CONTENTS.

ORIGINAL COMMUNICATIONS—

A SKETCH OF THE LIFE AND CHARACTER OF DR. H. V. M. MILLER. By Hooper Alexander ........ 289
ONE HUNDRED AND SIXTY-SIX CASES OF CANCER OF THE PREGNANT UTERUS OCCURRING SINCE 1886. By George H. Noble, M.D., Atlanta, Ga........ 297
PRACTICAL PATHOLOGY OF THE MUCOUS MEMBRANE OF THE NOSE, THROAT, AND EARS OF YOUTHS. By Thomas F. Rumbold, M.D., St. Louis, Mo........ 315
ON BONY GROWTHS INVADING THE TONSILS. By Alex. W. Stirling, M.D., (Hon.) M.B., C.M. (Edin.), D.P.H. (Lond.), Atlanta, Ga........ 328
COAL TAR ANTIPYRETICS. By J. A. Martin, M.D., Indianapolis, Ind........ 331

SOCIETY REPORTS—

ATLANTA SOCIETY OF MEDICINE........ 335

EDITORIAL—

ANNOUNCEMENT........ 347
JENNER........ 347

SELECTIONS AND ABSTRACTS—

GENERAL MEDICINE AND THERAPEUTICS........ 351

MEDICAL ITEMS........ 454
BOOK REVIEWS........ 359
MEDICAL ITEMS—CONTINUED........ x, xi, xii, xiii
ADVERTISERS' INDEX TO ADVERTISEMENTS........ xiv

